# Key-Point Detection Algorithm of Deep Learning Can Predict Lower Limb Alignment with Simple Knee Radiographs

**DOI:** 10.3390/jcm12041455

**Published:** 2023-02-11

**Authors:** Hee Seung Nam, Sang Hyun Park, Jade Pei Yuik Ho, Seong Yun Park, Joon Hee Cho, Yong Seuk Lee

**Affiliations:** Department of Orthopaedic Surgery, Seoul National University College of Medicine, Seoul National University Bundang Hospital, Seongnam-si 13620 82, Republic of Korea

**Keywords:** knee, weight-bearing line, machine learning, convolutional neural network, prediction

## Abstract

(1) Background: There have been many attempts to predict the weight-bearing line (WBL) ratio using simple knee radiographs. Using a convolutional neural network (CNN), we focused on predicting the WBL ratio quantitatively. (2) Methods: From March 2003 to December 2021, 2410 patients with 4790 knee AP radiographs were randomly selected using stratified random sampling. Our dataset was cropped by four points annotated by a specialist with a 10-pixel margin. The model predicted our interest points, which were both plateau points, i.e., starting WBL point and exit WBL point. The resulting value of the model was analyzed in two ways: pixel units and WBL error values. (3) Results: The mean accuracy (MA) was increased from around 0.5 using a 2-pixel unit to around 0.8 using 6 pixels in both the validation and the test sets. When the tibial plateau length was taken as 100%, the MA was increased from approximately 0.1, using 1%, to approximately 0.5, using 5% in both the validation and the test sets. (4) Conclusions: The DL-based key-point detection algorithm for predicting lower limb alignment through labeling using simple knee AP radiographs demonstrated comparable accuracy to that of the direct measurement using whole leg radiographs. Using this algorithm, the WBL ratio prediction with simple knee AP radiographs could be useful to diagnose lower limb alignment in osteoarthritis patients in primary care.

## 1. Introduction

Osteoarthritis (OA) is the most common form of arthritis, affecting millions of people worldwide [1]. It occurs when the protective cartilage that cushions the ends of the bones wears down [2]. Therefore, weight-bearing joints, such as the knee joint, are more vulnerable. The medial compartment of the knee joint is the most commonly affected site in OA, and the medial joint space narrows as OA progresses. This induces varus deformity of the lower limb. Consequently, the adduction moment, which is the magnitude of the ground reaction force, moves medially from the center of the knee joint during ambulation, and the moment arm of the ground reaction force increases [3]. In this manner, the varus deformity enters a vicious cycle. Therefore, it is important to intervene through treatment before such vicious cycle develops [4,5].

The evaluation of the weight-bearing axis of the lower limb is a fundamental step in the identification, classification, and treatment of lower limb deformities, which may result from degeneration, trauma, inflammation, or congenital diseases. When deciding between treatment options, such as conservative treatment, osteotomy, and arthroplasty, the weight-bearing axis should be considered in addition to the patient’s basic demographics [6]. Several methods assess the weight-bearing axis of the lower limbs. Techniques to determine the hip–knee–ankle angle, mechanical axis, and weight-bearing line (WBL) ratio are commonly used methods [7,8]. These parameters are usually measured on whole leg radiographs (WLR) and in institutions, such as large hospitals or community clinics [9]. Therefore, they may not be readily available because of the high costs involved. Moreover, primary physicians may face difficulties in identifying such deformities, and several attempts have been made to predict the WBL ratio using simple standing knee radiographs [10].

The application of artificial intelligence in medicine has gained popularity in recent years because of its ability to improve the efficiency of healthcare delivery and patient diagnosis. Karnuta et al. used machine learning to identify knee arthroplasty implants from X-rays [11]. In addition, Joseph et al. employed machine learning to forecast the development of osteoarthritis over 8 years using combined MR imaging features, demographics, and clinical factors as input [12]. Convolutional neural networks (CNNs) are a subtype of deep learning (DL) that have shown impressive outcomes in image classification and recognition [13,14]. A CNN was used in a previous study that predicted the WBL ratio as a parameter for lower limb alignment in simple knee radiographs. However, a limitation of this study was that the prediction was only possible within intervals. Therefore, a quantitative assessment that can predict the WBL ratio and translate it to an accurate point on the tibial plateau may be more intuitive for clinical use [15].

This study aimed to develop a DL algorithm to predict the point at which the weight-bearing axis of the lower limb crosses the tibial plateau. The hypothesis of this study was that the WBL ratio obtained from weight-bearing WLR could be predicted by a specially designed DL model using standing simple knee anteroposterior (AP) radiographs with high predictive value.

## 2. Materials and Methods

With the assumption that standing simple knee radiographs are a part of WLR and may be related to the lower limb alignment, the most appropriate and accurate model to predict the WBL ratio after learning the WBL ratio was designed using simple knee radiographs. This was based on the key-point detection model [16,17]. The key-point detection model involves locating the key object parts that represent the underlying object in a feature-rich manner. After directly labeling the WLR picture according to how we draw the WBL in a real clinical situation, we cut it into a simple knee radiograph picture and trained the DL model. Subsequently, the accuracy of the learned DL algorithm for measuring the WBL ratio was investigated. For the analysis of the accuracy of the DL model, its mean absolute error (MAE) and intra-class correlation coefficients (ICC) were evaluated. In addition, the accuracy values of the DL model were compared with the ICCs of the rater, using real measurements of the WBL ratio in WLR to check whether the DL algorithm was reliable as the measurements of the rater. All procedures involving human participants were performed following the ethical standards of the institutional review board (IRB No. B-2210-784-101) and the Helsinki Declaration (1964) and its later amendments. Consent was not sought because this study retrospectively reviewed the medical record image data of patients who underwent X-ray examination at our hospital, and personal identification information was not included in the data analysis process. Therefore, it was difficult to evaluate if the risk for the patients included in this study was increased compared to that of other patients. There is no reason to presume the refusal of consent.

### 2.1. Data Set

From March 2003 to December 2021, 89,709 patients with knee pain and standing knee AP radiographs were obtained from the clinical database of our hospital. Among them, 3515 patients (3.9% of standing knee AP acquisition) who underwent weight-bearing WLR were included. The exclusion criteria were as follows: (1) previous ipsilateral surgery (hip, knee, or ankle joint); (2) children with remaining growth plates; and (3) patients with deformity due to previous trauma or congenital diseases. After excluding patients who met the exclusion criteria, finally, 2410 patients with 4790 knee AP radiographs were randomly selected using stratified random sampling. To avoid the cluster effect between multiple radiographs in a single patient, only the initial knee AP radiograph was used.

### 2.2. WBL Ratio Measurement and Labeling

Our dataset was created by one specialist, and the data were cropped by four points annotated by a specialist (tibial plateau at both ends, WBL starting and exit points) with a 10-pixel margin. Although four points varied on the cropped data, and the data size was variable, the data were resized uniformly in the training phase. Therefore, our model was invariant in relation to the image size to some degree. The model’s robustness of the data size was evaluated with various random margins of 5 to 10 pixels. This experiment showed that the WBL ratio did not affect the data size, and our model predicted the line’s tendency. This revealed that even if the users crop the image data abnormally, the model’s prediction result will not decline.

The WBL ratio was measured using the weight-bearing WLR of all 2410 patients with 4790 knees for labeling the training set and analysis of prediction accuracy in the validation and test sets. The WBL was drawn from the center of the femoral head to the center of the superior articular surface of the talus. The WBL ratio was calculated as the ratio of the crossing point of the mechanical axis, from the medial edge to the entire width of the tibial plateau.

### 2.3. Image Preprocessing

A standing knee AP radiograph was chosen as the research object. The PyDicom library (version 1.3.0) was used for the preprocessing of DICOM images. The right or left knee was cropped in a knee radiograph that included both knees. Strong augmentation, such as sheer, distortion, and high rate of random brightness, contrast, equalization, and hue saturation for robustness, was used to improve the performance of the algorithm.

### 2.4. DL Algorithm

**Figure 1:**
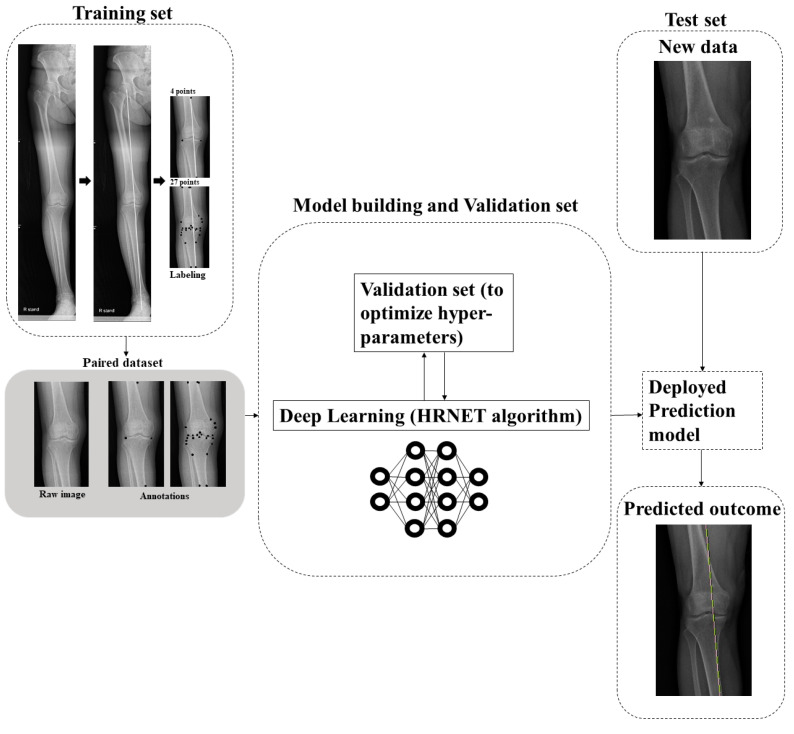
Pipeline of the study. Annotations: labeled image. Training set: the data sample used to fit the model. Validation set: the data sample used to provide an unbiased evaluation of a model fit on the training dataset while tuning the model hyperparameters. Test set: the data sample used to provide an unbiased evaluation of the final model fit on the training dataset.

The DL algorithm consists of two stages. In the first stage, key-points heat maps were predicted using the WBL prediction model. Our WBL prediction model is similar to conventional pose estimation, which predicts some key-points through heat maps and is composed of a feature extractor and a simple convolutional decoder. The model predicts our points of interest, which are both plateau points, i.e., the starting WBL point and the exit WBL point. Using the logits calculated from the model, we trained our model in an end-to-end manner with the Adam W optimizer and binary cross entropy loss. The other setting was similar to that of the conventional DL. In the second stage, the WBL ratio was calculated using the four predicted points. Two lines from four points were drawn to determine the intersection of the two lines and calculate the WBL ratio (Figure 1).

### 2.5. Experiment

In the experiment, our model for 10,000 iterations using Adam W with β1 = 0.9, β2 = 0.999 was trained; learning rate = 1 × 10^−3^, weight decay = 1 × 10^−2^, cosine decaying scheduler, and binary cross entropy loss. In the training phase, the cropped image data were transformed using random brightness, contrast, equalization, and hue saturation for robustness. Our model was on four 2080 ti GPU with an 8-batch size per GPU. The resulting value of the model was analyzed in two ways: WBL error values and pixel units. When analyzing the results with the WBL error value, a value of 1 was assigned if the DL prediction value was within each error value of the tibial plateau, and a value of 0 was assigned otherwise. When analyzing the results in pixel units, the accuracy was calculated by assigning a value of 1 if the DL prediction was received in each pixel unit, and a value of 0 otherwise. The algorithm was first trained by making as many landmarks as possible, i.e., 27 dots, around the knee joint. According to the traditional WBL calculation method, radiological landmarks that can symbolize the bony anatomy of both femur and tibia were used, so there were 27 of them, as follows. The starting point and exit point of the WBL line, both endpoints of the femur cortex in X-ray images, both endpoints of the tibia cortex in X-ray images, both endpoints of femur and tibia at the tibiofemoral joint—the most distant points medial and lateral from femur and tibia and transepicondyle points at the tibiofemoral joint—trochlear notch center—midpoint of both tibial spines and tibial spines, intersection of tibia and fibula, inflection points at femur and tibia. The marking was reduced by identifying the most appropriate dots that showed the best performance (Figure 1).

### 2.6. Statistical Analysis

Data are presented as means and standard deviations for continuous variables. One-way analysis of variance was performed to compare the quantitative variables (i.e., age, body mass index (BMI), and WBL ratio). Pearson’s chi-squared test or Fisher’s exact test was used to compare the qualitative variables (i.e., gender). Statistical significance was set at *p* < 0.05. The data were analyzed using SPSS 25.0 (IBM, Armonk, NY, USA). To examine the reproducibility of the calculation of the WBL ratio using WLR, two observers were chosen: A, an orthopedic surgeon with 5 years of experience; B. an orthopedic surgeon with 20 years of experience. Independent measurements obtained by each of the two raters for each data set (raters A, B) and two independent measurements (A1 and A2) obtained by a single rater for each data set were compared. The mean difference between the independent measurements obtained by the raters was evaluated. The inter- and intra-observer reliabilities of the measurements were analyzed using ICC, with ICC < 0.40 indicating poor agreement, in the range 0.40–0.75 indicating fair to good (moderate) agreement, and in the range 0.76–1.00 indicating excellent agreement. MAE was used as a measure to determine how well the CNN fit the WBL ratio [13,14,15]. MAE is a measure that indicates the difference between the actual labeled WBL ratio by A (AL) using WLR and the WBL ratio predicted by the CNN using simple knee radiographs, $MAE=\frac{1}{N}\overset{N}{\underset{i=1}{\sum }}\left|\hat{y_{i}}-y_{i}\right|$, with $\hat{y_{i}}$ being the estimated WBL ratio of the ith data, and $y_{i}$ being the ground-truth WBL ratio of the ith data [18].

## 3. Results

**Figure 2:**
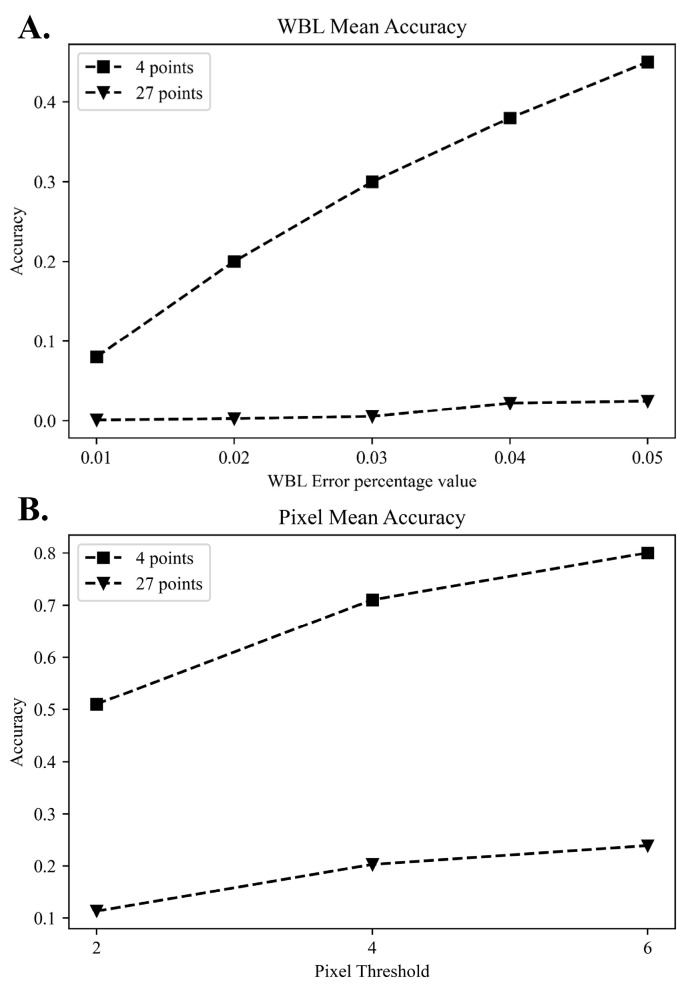
Comparison of WBL mean accuracy in 4 and 27 points. (**A**) Comparison graph of 4 and 27 points in WBL error percentage. (**B**) Comparison graph of 4 and 27 points in pixel threshold. Validation set: the data sample used to provide an unbiased evaluation of a model fit on the training dataset while tuning the model hyperparameters. Test set: the data sample used to provide an unbiased evaluation of a final model fit on the training dataset.

**Figure 3:**
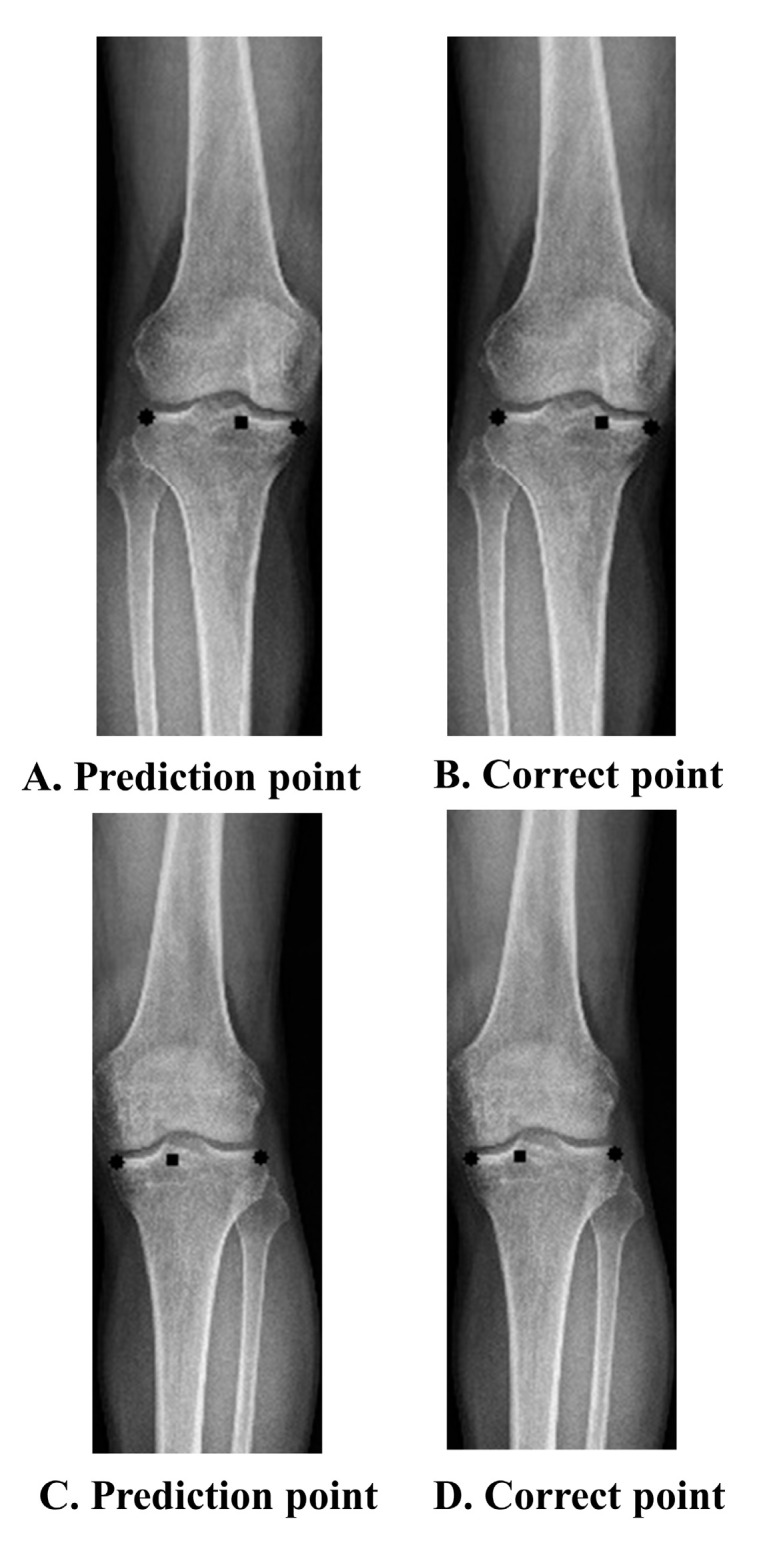
Prediction and correct point. (**A**–**D**) Prediction point vs. correct point. (**A**) Prediction point in Rt knee, (**B**) correct point in Rt knee, (**C**) prediction point in Lt knee, (**D**) correct point in Lt knee. Prediction point: point predicted by the deep learning algorithm. Correct point: labeled point.

The baseline characteristics of the patients and the distribution of the labels in the training, validation, and test sets are summarized in Table 1. Age, sex, BMI, and WBL ratio were not significantly different between the datasets. The performance was improved through simple labeling that marked both ends of the tibia and the starting and exit points of the WBL line. Four points indicated the best performance among the trials. A comparison of the WBL mean accuracy at 4 and 27 points is shown in Figure 2. As the threshold of the WBL error percentage value increased, the accuracy of taking four points approached 0.6, whereas when 27 points were taken, a steady state was reached with an accuracy not exceeding 0.1. These results were also similarly obtained in the pixel unit, and as the threshold increased, the accuracy when taking four points was close to 0.8 in 6 pixels, while that achieved when taking 27 points was approximately 0.2 in 6 pixels. A representative example of a patient’s difference between the predicted and the correct points on an X-ray image is shown in Figure 3. The differences between the actual measured WBL ratio and the WBL ratio predicted by the CNN in this patient were 0.03 in the right knee and 0.05 in the left knee.

The prediction of the algorithm implemented by learning is shown in Table 2 using units of pixels and WBL error values. The mean accuracy (MA) was increased from around 0.5 using a 2-pixel unit to around 0.8 using 6 pixels in both the validation and the test sets. The probability of the prediction and target values entering within 6 pixels was close to 0.8. When the tibial plateau length was taken as 100%, the MA was increased from approximately 0.1, using 1%, to approximately 0.5, using 5%, in both the validation and the test sets. The probability of obtaining a value within 5% exceeded 0.5.

**Figure 4:**
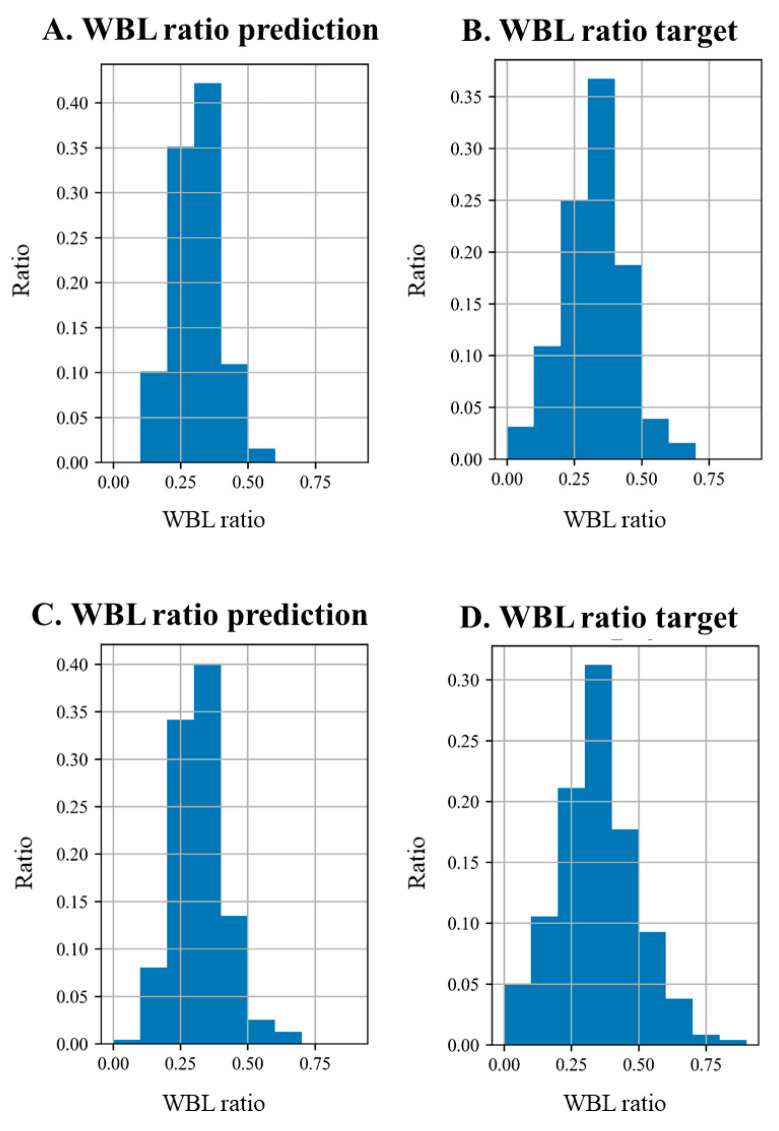
Histogram of the WBL ratio distribution in the validation and test sets. (**A**–**D**) Prediction distribution vs. target distribution. (**A**) WBL ratio prediction distribution in the validation set, (**B**) WBL ratio target distribution in the validation set, (**C**) WBL ratio prediction distribution in the test set, (**D**) WBL ratio target distribution in the test set. WBL ratio prediction: predicted value of the WBL, WBL ratio target: calculated WBL target.

**Figure 5:**
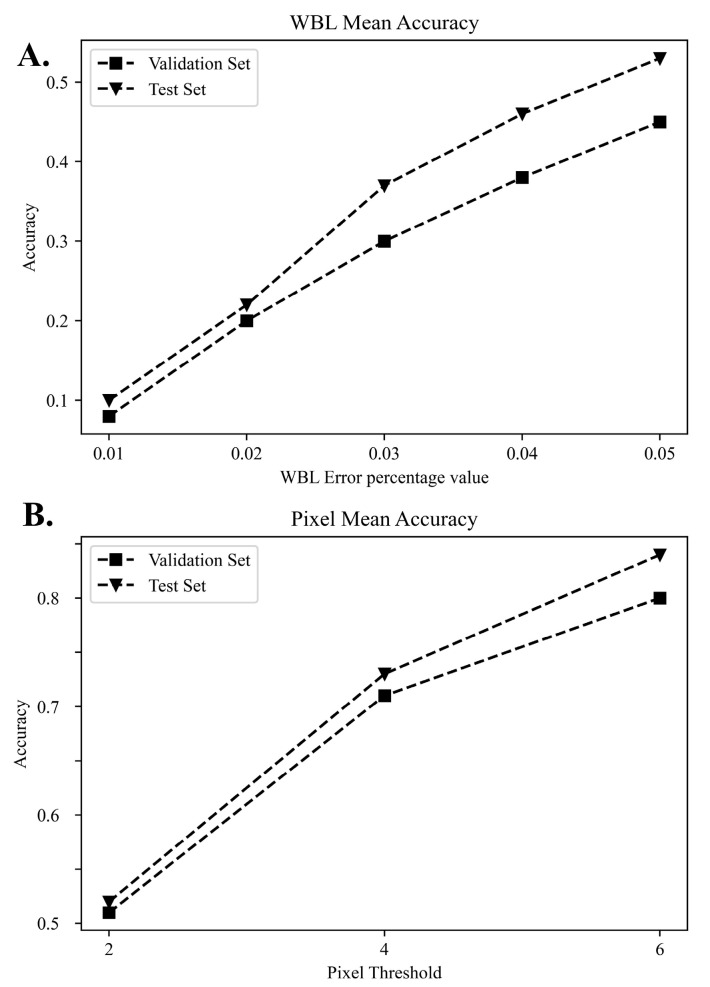
Graph of the WBL mean accuracy. (**A**) Accuracy graph of the WBL error percentage value, (**B**) accuracy graph of the pixel threshold. Validation set: the data sample used to provide an unbiased evaluation of a model fit on the training dataset while tuning the model hyperparameters. Test set: the sample of data used to provide an unbiased evaluation of a final model fit on the training dataset.

The mean difference, ICC, and MAE value are shown in Table 3. The mean difference of the validation and test sets between intra-observer and inter-observer measurements of the WBL ratio using a WLR ranged from 0.023 to 0.036. The MAE of the validation and test sets, with the DL model measuring the WLB ratio using simple knee radiographs were 0.064 (95% CI, 0.057–0.071) and 0.051 (95% CI, 0.044–0.058), respectively. The ICCs of the validation and test sets were all over 0.8, which indicated excellent agreement. The distributions of the WBL predictions in the validation and test sets are shown in Figure 4. The distribution of the DL model showed less difference in the high incidence area, where the WBL ratio was between 0.25 and 0.50. However, higher differences in the section that showed a lower incidence of the WBL ratio were noted. These values were similar for both the validation and the test sets. The MA of the WBL ratio for the validation and test sets is shown in Figure 5. As the thresholds of the WBL error percentage value and pixel units increased, the accuracy increased in both the validation and the test sets. The accuracy increased from around 0.1 to 0.5 with the increase in the WBL error percentage value thresholds, and from around 0.5 to 0.8 with the increase in the pixel threshold.

## 4. Discussion

The principal finding of this study is that the novel approach using the four-point marked key-point detection algorithm could predict the alignment of the lower limb using standing knee AP radiographs with high accuracy, comparable to that achieved with the direct measurement of the WLR. As one pixel was 0.265 mm, approximately 80% of the test and validation set prediction values were entered into 6 pixels. Therefore, it was assumed that approximately 80% of patients could be correctly evaluated within approximately 1 mm intervals.

The mean difference, MAE, and ICC values were used to test the accuracy and reliability of this study [19]. The accuracy of DL was indirectly estimated by comparing the mean difference between the values measured by the raters using WLR and the MAE of the values of DL predicting the WBL ratio using simple knee radiographs. The WBL ratio showed a slightly increase in the MAE when compared with the mean difference, because the WLR was not provided. In addition, the ICC of DL was also lower than the ICC of the values measured by the raters using WLR, but we observed that it provided a relatively good prediction at 0.8 or more.

Several trials to predict lower limb alignment using simple knee radiographs by linear regression analysis exist [10,20]. However, the results are not satisfactory, and to obtain satisfactory results, an X-ray image approximately 20 cm in length above and below the knee joint was required [21]. To solve these problems, a new prediction method based on DL using a key-point detection algorithm is proposed in our study. The key-point detection algorithm was considered the most suitable model because of the ability of the algorithm to find a specific point where the WBL passes through the tibia plateau [22,23]. Key-point detection algorithms are often used for pose estimation, face detection, and object detection [17,23]. Interestingly, the prediction accuracy decreased when the marking increased. This seems to be due to the characteristics of the DL model. DL is so automatic and high dimensional that the process of calculating the output by extracting features from the input is represented as a black box [13,24,25]. Therefore, it can be understood that if more labeling is performed, the labeling error increases, and the automatic feature extraction process is hindered; thus, the prediction accuracy could be lowered, as occurred in our study.

From the patient’s point of view, visiting a tertiary hospital for WLR is time-consuming, expensive, and leads to high radiation exposure [26]. If the WBL ratio can be predicted through simple knee AP radiographs using this algorithm in primary care, it will be possible to easily determine the treatment process, as well as the degree of arthritis in more detail. This will also be useful for follow-up evaluation of patients who underwent re-alignment procedures such as osteotomy [19,27]. Expecting lower limb alignment using only simple knee radiographs has a lot of pros in the decision of patient-specific treatment protocols in various kinds of institutions. A DL model for predicting the WBL ratio through a simple knee radiograph was not attempted in the past, but it will become an essential medical technique in the future society characterized by the use of artificial intelligence [28].

The strength of this study is that a more accurate prediction of lower limb alignment can be obtained using the DL key-point detection model. Our study is meaningful in that it not only uses a key-point detection model, but also takes a significant point and trains ML to make a prediction using this model. In addition, this study has significance as it allows predicting the WBL ratio using a simple knee AP radiograph in a situation where WLR imaging is limited. Primary care physicians can properly diagnose patients with knee OA using the DL model. In addition, it is expected that planning the realignment procedure would be possible with high accuracy.

This study has several limitations. First, it was difficult to interpret the developed CNN model itself; therefore, it was hard to determine whether the CNN model focused on the WBL prediction. Second, the number of test sets was small, although the ratio between the validation and the test sets was adequate. Third, the WBL predictions of our model were relatively distributed around the center compared with the target WBL value. Thus, our model was trained on general WBL values and has limitations in predicting infrequent WBL values. This is because of the small amount of WBL data corresponding to outliers, as shown in Figure 4. This phenomenon is expected to decrease as the amount of data increases. Fourth, the prediction would be inaccurate if there is a deformity in the proximal femur or distal tibia, because this cannot be checked on standing simple knee radiographs. Fifth, since the study was conducted on patients who presented to tertiary medical institutions, there may be a selection bias in the patient group. This is because these patients were referred from primary and secondary medical institutions. The severity of disease in patients visiting tertiary care may be higher than that of patients visiting primary or secondary health care institutions.

## 5. Conclusions

The DL-based key-point detection algorithm to predict lower limb alignment through labeling using simple knee AP radiographs demonstrated comparable accuracy to that of the direct measurement using whole leg radiographs. Using this algorithm, the WBL ratio prediction with simple knee AP radiographs could be useful to diagnose lower limb alignment in osteoarthritis patients in primary care.

## Figures and Tables

**Table 1:** Baseline characteristics of the dataset.

	Training Set	Validation Set	Test Set	Total	*p*-Value
Age (year)	65.1 ± 9.31	65.0 ± 9.42	65.4 ± 9.27	65.2 ± 12.2	0.521
Gender (M/F)	391/1928	46/241	49/241	486/2410	0.215
BMI (kg/m^2^)	25.7 ± 3.22	25.8 ± 2.51	25.3 ± 2.37	25.7 ± 3.21	0.333
WBL ratio	0.33 ± 0.17	0.31 ± 0.15	0.32 ± 0.12	0.32 ± 0.15	0.087

Values are presented as number or mean ± standard deviation. M: men, F: female, BMI: body mass index, WBL: weight-bearing line. Training set: the sample of data used to fit the model. Validation set: the sample of data used to provide an unbiased evaluation of a model fit on the training dataset while tuning the model hyper-parameters. Test set: the sample of data used to provide an unbiased evaluation of a final model fit on the training dataset.

**Table 2:** Results regarding the prediction of the deep learning algorithm.

	Validation Set					Test Set				
Pixel	2 pixels		4 pixels	6 pixels		2 pixels		4 pixels	6 pixels	
MA	0.51 ± 0.07		0.71 ± 0.06	0.80 ± 0.08		0.52 ± 0.06		0.73 ± 0.06	0.84 ± 0.04	
WEV	1%	2%	3%	4%	5 %	1%	2%	3%	4%	5%
MA	0.08 ± 0.04	0.20 ± 0.05	0.30 ± 0.06	0.38 ± 0.07	0.45 ± 0.07	0.10 ± 0.05	0.22 ± 0.07	0.37 ± 0.09	0.46 ± 0.11	0.53 ± 0.11

Values are presented as number or mean ± standard deviation. MA: Mean accuracy, WEV: WBL error value. Validation set: the sample of data used to provide an unbiased evaluation of a model fit on the training dataset while tuning model hyper-parameters. Test set: the sample of data used to provide an unbiased evaluation of a final model fit on the training dataset.

**Table 3:** Mean difference, mean absolute error, and ICC in the validation and test sets.

Validation Set	Mean Difference	ICC
A1 and A2	0.023 ± 0.005	0.96 ± 0.02
A1 and B	0.034 ± 0.003	0.94 ± 0.05
Test set	Mean difference	ICC
A1 and A2	0.024 ± 0.006	0.97 ± 0.03
A1 and B	0.036 ± 0.004	0.95 ± 0.05
Validation set	Mean absolute error	ICC
AL and DL	0.064 ± 0.007	0.89 ± 0.09
Test set	Mean absolute error	ICC
AL and DL	0.051 ± 0.007	0.88 ± 0.08

Values are presented as number or mean ± standard deviation. ICC, intra-class correlation coefficients. Validation set, the sample of data used to provide an unbiased evaluation of a model fit on the training dataset while tuning the model hyper-parameters. Test set, the sample of data used to provide an unbiased evaluation of a final model fit on the training dataset; A1, rater 1, A2: rater 1 at different times; B, rater 2; AL, rater 1 with labeling on the WLR; DL, deep learning.

## Data Availability

Y.S.L. (smcos1@hanmail.net) had full access to all of the data in the study and takes responsibility for the integrity of the data and the accuracy of the data analysis. All authors have read and agreed to the published version of the manuscript.
